# Complete mitochondrial genome and the phylogenetic position of the Leadhued skate *Notoraja tobitukai* (Rajiformes: Arhynchobatidae)

**DOI:** 10.1080/23802359.2016.1192514

**Published:** 2016-10-29

**Authors:** Ranran Si, Xiao Chen, Hao Chen, Weiming Ai

**Affiliations:** a Department of Marine Science, Wenzhou Medical University, Wenzhou, Zhejiang, PR China;; b College of Marine Science, South China Agriculture University, Guangzhou, Guangdong, China

**Keywords:** Arhynchobatidae, *Notoraja tobitukai*, Rajiformes

## Abstract

In this study, the complete mitochondrial genome of the Leadhued skate *Notoraja tobitukai* (Rajiformes: Arhynchobatidae) was determined. It was a circle molecular (16, 799 bp), consisted of 37 genes with a typical gene order in vertebrate mitogenome. In the whole mitogenome, there were 24 bp short intergenic and 26 bp overlaps. The nucleotide composition was 32.3% A, 22.6% C, 13.1% G and 32.0% T. Two start codons (GTG and ATG) and two stop codons (TAG, TAA/T) were used in the protein-coding genes. The 22 tRNA genes ranged from 65 bp (tRNA-*Cys*) to 75 bp (tRNA-*Leu1*). The phylogenetic result showed that *N. tobitukai* was clustered with the *Pavoraja nitida*.

TheLeadhued skate *Notoraja tobitukai* was distributed in the Northwest Pacific, from Japan and Okinawa Trough to East China Sea and Taiwan (McEachran & Dunn 1998). This deep sea species were oviparous, which eggs were oblong capsules with stiff pointed horns at the corners deposited in sandy or muddy flats (Breder & Rosen 1966). In this study, we determined the complete mitochondrial genome of *N. tobitukai* for the first time and analyzed the phylogenetic relationship in Rajiformes.

One specimen of *N. tobitukai* was captured from South China sea and preserved in the Museum of Marine biology in Wenzhou Medical University (voucher: NH2011071608). The experimental protocol and data analysis methods followed Chen et al. (2015). Including *N. tobitukai*, thirteen species of Rajiformes with the complete mitogenomes available in the Genbank, were selected to construct the phylogenetic tree by using Bayesian method. The outgroups were *Dasyatis akajei* and *D. bennetti* (Myliobatoformes).

The complete mitochondrial sequence of *N. tobitukai* was 16, 799 bp (GenBank accession KX150853), consisting of 13 protein-coding genes, 2 rRNAs, 22 tRNAs, and a noncoding control region, with the gene order identical to that of typical vertebrates. The nucleotide composition was: 32.3% A, 32.0% T, 22.6% C and 13.1% G. The A + T content (64.3%) was higher than the G + C content (35.7%). Its whole mitogenome had 24 bp short intergenic spaces located in 12 gene junctions and 26 bp overlaps located in 7 gene junctions. The 13 protein-coding genes used two start codons (GTG and ATG) as well as two stop codons (TAG and TAA/T), and most of them shared common initial codon ATG and terminal codon TAA/T. The *COI* gene owned a nonstandard initial codon GTG, which was common in vertebrates (Slack et al. 2003). The *COII* and *ND4* genes were terminated with a single T, which could be extended to complete TAA through polyadenylation in transcriptions (Ojala et al. 1981). Both 12S rRNA (965 bp) and 16S rRNA (1, 677 bp) genes were between tRNA-*Phe* and tRNA-*Leu1* genes, separated by tRNA-*Val* gene. A noncoding sequence (30 bp) associated with the putative L-strand replication origin (OL) located between tRNA-*Asn* and tRNA-*Cys* in the WANCY. The control region was 1,154 bp, presenting a high A + T content (70.5%).

Within the Rajiformes, thirteen available species were divided into three families with the (Rhinobatidae + (Rajidae + Arhynchobatidae)) relationship, which were consistented to the morphological result (McEachran & Dunn 1998). The topology showed that three families and all genuses were monophyletic. *Notoraja tobitukai* was sister to the *Pavoraja nitida* clade, then this clade clustered to *Atlantoraja castelnaui* (Figure 1).

**Figure 1.**
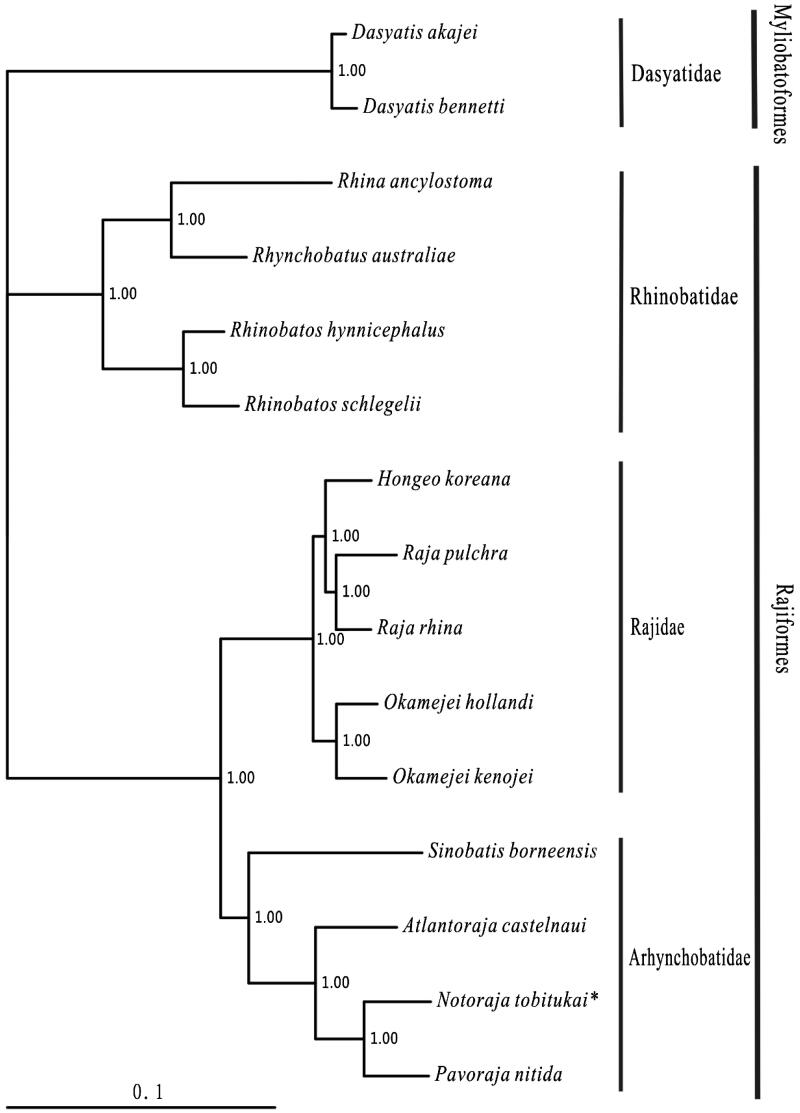
Phylogenetic position of *Notoraja tobitukai*. *Dasyatis akajei* (NC_021132.1) and *D. bennetti* (KC633222.1) were selected as the outgroup. The thirteen species of Rajiformes were *Rhina ancylostoma* (KU721837), *Rhynchobatus australiae* (KU746824), *Rhinobatos hynnicephalus* (NC_022841.1), *R. schlegelii* (NC_023951.1), *Hongeo koreana* (NC_021963.1), *Raja pulchra* (NC_025498.1), *R. rhina* (KC914434.1), *Okamejei hollandi* (KP756687.1), *O. kenojei* (NC_007173.1), *Sinobatis borneensis* (KX014715), *Atlantoraja castelnaui* (NC_025942.1), *Notoraja tobitukai* (KX150853) and *Pavoraja nitida* (NC_024599.1).
